# Albiflorin on Neuropsychiatric and Neurodegenerative Disorders: A Systematic Review

**DOI:** 10.1111/cns.70535

**Published:** 2025-07-25

**Authors:** Shasha Sun, Hamizah Shahirah Hamezah, Chuanshan Jin, Rongchun Han, Xiaohui Tong

**Affiliations:** ^1^ School of Pharmacy Anhui University of Chinese Medicine Hefei China; ^2^ Institute of Systems Biology Universiti Kebangsaan Malaysia Bangi Malaysia; ^3^ AHUCM‐UKM Joint Laboratory for Traditional Medicine Quality Standardization Anhui University of Chinese Medicine Hefei China; ^4^ Joint Research Center for Chinese Herbal Medicine of Anhui of IHM Bozhou Vocational and Technical College Bozhou China; ^5^ School of Life Sciences Anhui University of Chinese Medicine Hefei China

**Keywords:** albiflorin, Alzheimer's disease, mental disorder, pharmacokinetics

## Abstract

**Aims:**

Albiflorin, a key compound from *Paeonia lactiflora*, has shown therapeutic potential in neuropsychiatric and neurodegenerative disorders (NPDs and NDDs), especially depression and Alzheimer's disease (AD). This review aimed to summarize its pharmacological effects, mechanisms, pharmacokinetics, and therapeutic prospects.

**Discussion:**

Albiflorin exhibits multi‐target actions, including modulation of monoamine neurotransmitters, inhibition of neuroinflammation, and enhancement of neuroplasticity. In AD, it reduces Aβ accumulation, improves mitochondrial function, and activates MAPK/ERK and Nrf2/HO‐1 signaling pathways. In depression, it restores phospholipid and tryptophan metabolism, regulates HPA axis function, and increases BDNF expression. Albiflorin crosses the blood‐brain barrier (BBB) and may act indirectly via the gut‐brain axis through its metabolite benzoic acid. Though brain concentrations are low, its pharmacological effects remain significant. Albiflorin also shows potential benefits in conditions like cerebral ischemia and hypoxic‐ischemic brain injury. Toxicological data indicate low systemic toxicity and good safety margins *in vivo* and *in vitro*.

**Conclusions:**

Albiflorin demonstrates promising therapeutic potential for NPDs and NDDs via multi‐pathway regulation. However, further studies are needed to optimize brain delivery, understand gut microbiota interactions, and confirm efficacy through clinical trials. The advancement of formulation strategies and pharmacokinetic research will be considered key to achieving clinical translation.

## Graphical abstract

This review summarizes that albiflorin alleviates depression by modulating neuroinflammation and enhancing neurotrophic signaling. Following oral administration, albiflorin crosses the blood‐brain barrier and acts primarily on the hippocampus and prefrontal cortex. It suppresses microglial activation and reduces the expression of pro‐inflammatory cytokines such as IL‐1β and TNF‐α. At the same time, albiflorin promotes the expression of brain‐derived neurotrophic factor (BDNF) through the activation of key signaling pathways, including PI3K/Akt, MEK/ERK, and cAMP/PKA. These molecular effects contribute to neuronal repair, restoration of synaptic plasticity, and the mitigation of depressive‐like behaviors. Altogether, the findings highlight the multi‐target mechanisms through which albiflorin exerts its antidepressant effects, supporting its promise as a potential therapeutic agent for neuropsychiatric disorders.

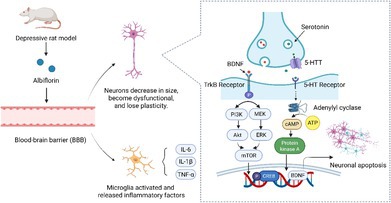

**Abbreviations**
%OAE: percentage of open arm entries
%TOA: percentage of time spent in open arms
5‐HIAA: 5‐hydroxyindoleacetic acid
5‐HT: serotonin
5‐HTT: serotonin transporter
ACE: angiotensin‐converting enzyme
ACTH: adrenocorticotropic hormone
AD: Alzheimer's disease
AKT/PI3K: serine–threonine kinase/phosphoinositide 3‐kinase
Ang‐1: angiogenin‐1
ASC: apoptosis‐associated speck‐like protein containing a caspase recruitment domain
AUC_0–∞_
: total area under the curve
AUC_last_
: area under the plasma concentration–time curve from zero to the last measurable concentration
Aβ: amyloid β‐protein
BA: benzoic acid
BBB: blood–brain barrier
BDNF: brain‐derived neurotrophic factor
cAMP/PKA: cyclic adenosine monophosphate/protein kinase A
cGMP: cyclic guanosine monophosphate
CHSGS: Chaihu Shugan San
CI: cerebral ischemia
*C*_max_
: maximum concentration
cPLA2: cytosolic phospholipase A2
CREB: cAMP‐response element binding protein
CUMS: chronic unpredictable mild stress
CUS: chronic unpredictable stress
DAAO: d‐amino acid oxidase
DSS: Danggui‐Shaoyao‐San
ELISA: enzyme‐linked immunosorbent assay
ERK: extracellular signal‐regulated kinase
FST: forced swimming test
GluR1: glutamate receptor 1
GPX4: glutathione peroxidase 4
GR: glucocorticoid receptor
H_2_O_2_
: hydrogen peroxide
HE: hematoxylin–eosin
HIF‐1α: hypoxia‐inducible factor‐1α
HPA: hypothalamic–pituitary–adrenal
HTRP: Huatuo Reconstruction Pill
Iba1: ionized calcium binding adaptor molecule 1
IDO1: indoleamine 2,3‐dioxygenase 1
IL‐1β: interleukin‐1β
IL‐6: interleukin‐6
IL‐9: interleukin‐9
IR: ischemia–reperfusion
IRF7: interferon regulatory factor 7
LDH: lactate dehydrogenase
MAPK: mitogen‐activated protein kinase‐1
MCAO: middle cerebral artery occlusion
MEK: mitogen‐activated extracellular signal‐regulated kinase
MLV: meningeal lymphatic vessel
mTOR: mammalian target of the rapamycin
MTT: 3‐(4,5‐dimethylthiazol‐2‐yl)‐2,5‐diphenyltetrazolium bromide
NDDs: neurodegenerative disorders
NE: norepinephrine
NF‐κB: nuclear factor‐kappa B
NLRP3: nucleotide‐binding oligomerization domain‐like receptor protein 3
nNOS: nitric oxide synthase
NO: nitric oxide
NPDs: neuropsychiatric disorders
Nrf2/HO‐1: nuclear factor erythroid 2‐related factor 2/heme oxygenase 1
OBX: olfactory bulbectomy
OFT: open field test
OGD/R: oxygen–glucose and deprivation/reoxygenation
PCNA: proliferating cell nuclear antigen
PGF: pseudo‐germ‐free
PGK1: phosphoglycerate kinase 1
PRRE: Paeoniae Radix Rubra extract
qRT‐PCR: quantitative real‐time PCR
ROS: reactive oxygen species
sAD: sporadic Alzheimer's disease
sGCα2: guanylate cyclase 1 soluble subunit alpha 2
SOD: superoxide dismutase
*T*_1/2_
: biological half‐life
TCA: tricarboxylic acid
*T*_max_
: time to maximum concentration
TNF‐α: tumor necrosis factor‐α
TrkB: tropomyosin receptor kinase B
TSPO: mitochondrial translocator protein 18 kDa
TST: tail suspension test
TXNIP: thioredoxin interacting protein
TXNIP: thioredoxin‐interacting protein
WB: Western blotting
WHO: World Health Organization

## 1 Introduction

Mental disorders (also called psychiatric disorders) are acknowledged as a variety of diseases that affect thinking, emotions, behavior, and cognitive functions, including neuropsychiatric disorders (NPDs) and neurodegenerative disorders (NDDs) as well. It was found that there is a complex interaction between NPDs and NDDs, both of which involve deteriorating neuronal cell death and behavioral changes [1]. NDDs, like Alzheimer's disease (AD), are progressive disorders marked by the degeneration and loss of neuronal function in the brain and spinal cord, leading to cognitive decline and memory loss. In contrast, NPDs, including depression and anxiety, involve both neurological and psychiatric symptoms, affecting the brain's structure, function, and chemical balance due to central nervous system abnormalities [2, 3]. The pathological factors of psychiatric disorders typically involve neurotransmitter imbalances, neuroinflammation, oxidative stress, among others, which can subsequently cause issues such as loss of motor function and autonomic dysfunction in individuals [4]. Statistics from the World Health Organization (WHO) showed that approximately 5% of adults worldwide were affected by depression each year [5]. In 2020, the Corona Virus Disease 2019 pandemic contributed to a global surge, with 5.32 million new depression cases and 76.2 million new anxiety cases, representing a notable increase in prevalence [6]. However, treating mental disorders can be tricky. In the present clinical application, Donepezil was frequently prescribed for different severities of AD and Triselyn for depression and anxiety, while patients often experience notable side effects and risks, such as drowsiness, dizziness, and psychological dependence. As the global prevalence of mental disorders continues to rise, there is a growing need for more effective medications with fewer side effects to treat these conditions in clinic.

Currently, the search for medications to treat mental disorders primarily focuses on neurotransmitter regulation, as well as targeting neuroinflammation, neural circuit modulation, and neuroplasticity [7, 8, 9, 10]. During the Eastern Han Dynasty, *Paeonia lactiflora* was documented in *Shennong's Classic of Materia Medica* (*Shennong Ben Cao Jing*) as having the ability to harmonize the liver and spleen [11]. It was traditionally combined with other Chinese herbs such as Bupleurum Radix (Chaihu) and Bupleuri Radix (Baizhu) in classic prescriptions, used for alleviating mood disorders for more than a millennium [12, 13]. Albiflorin is a bioactive compound derived from the dried root of *P. lactiflora* , exhibiting multi‐target pharmacological activities. It modulates several signaling pathways, including the NLRP3 inflammasome, Nrf2/HO‐1, MAPK, PI3K/Akt, and JAK/STAT, and has been widely studied in the context of NPDs and NDDs [14, 15, 16, 17]. Studies have shown that albiflorin can alleviate depression‐like behaviors by regulating neurotransmitters such as serotonin, dopamine, and norepinephrine, inhibiting neuroinflammation, and enhancing neuroplasticity, with a favorable safety profile [18]. As research into Chinese herbal medicine progresses, evidence shows that albiflorin affects the central nervous system and exhibits low toxicity [19, 20]. Although its direct distribution in brain tissue is limited, recent research suggests that albiflorin may exert indirect effects on brain function by modulating the gut microbiota and its metabolites (e.g., benzoic acid), aligning with the emerging concept of the gut–brain axis [21]. Compared to fluoxetine, albiflorin demonstrates better efficacy, fewer side effects, and efficient intestinal absorption, making it well‐suited for quantification, standardization, and pharmacokinetic analysis [22, 23]. Albiflorin has been widely studied in Asian countries such as China and South Korea with *P. lactiflora* traditionally used to treat diseases; however, reports on albiflorin from other regions are rare. Given its demonstrated therapeutic potential for mental disorders, albiflorin is expected to attract increasing attention from researchers worldwide, which would promote further mechanistic studies and subsequent medical applications [24]. This review seeks to explore the effects of albiflorin on mental disorders and the underlying mechanisms.

## 2 The Therapeutic Effects of Albiflorin on NDDs

Albiflorin excels in treating mental disorders by exhibiting anti‐inflammatory, neuroprotective, and antioxidant effects. These reported mechanisms offer a solid pharmacological foundation for the understanding of albiflorin on NDDs such as AD. This review compiles information on NDDs which are subcategorized into distinct diseases (Table 1), providing an overview of research on albiflorin's possible mechanisms of action.

**TABLE 1:** The therapeutic effects of albiflorin on NDDs and its possible mechanisms of action.

	Models	Mechanisms of action	References
AD	Sprague Dawley (SD) rats	Albiflorin demonstrates a protective effect against neurotoxicity caused by amyloid‐β (Aβ) species in primary hippocampal neuronal cells	[25]
AD	APP/PS1 mice	Albiflorin improves mitochondrial function, reduces brain Aβ accumulation, and alleviates memory impairments in mice	[26]
AD	SAMP1 and SAMP8 mice	Anti‐inflammatory effect of albiflorin is primarily achieved through neuroprotection and reducing microglial activation	[27]
Sporadic Alzheimer's disease (sAD)	C57BL/6 male mice	Albiflorin could be the key active substance in the aqueous extract of Danggui‐Shaoyao‐San (DSS) that regulates interferon regulatory factor 7 (IRF7) to improve meningeal lymphatic drainage of brain lymph and inhibit neuroinflammation in sAD	[28]
AD	PC 12 cells	Albiflorin's anti‐AD effects involve activation of the mitogen‐activated protein kinases/extracellular signal‐regulated kinase (MAPK/ERK) signaling pathway, which inhibits neuronal apoptosis	[29]
Cerebral ischemia (CI)	Male SD rats	Albiflorin protects against CI via modulating the phosphoglycerate kinase 1/the nuclear factor erythroid 2‐related factor 2/heme oxygenase 1 (PGK1/Nrf2/HO‐1) signaling pathway	[14]
CI	Male SD rats	Albiflorin protects against cerebral ischemia–reperfusion (IR) injury by enhancing antioxidant effects through increasing superoxide dismutase (SOD) and reducing lactate dehydrogenase (LDH)	[30]
CI	Male SD rats and HT22 cells	Albiflorin contributes to the regulation of ferroptosis and autophagy	[15]
CI	PC 12 cells and male SD rats	Albiflorin mitigates cerebral IR injury by modulating the nuclear factor erythroid 2‐related factor 2/heme oxygenase 1 (Nrf2/HO‐1) pathway	[31]

### 2.1 Alzheimer's Disease

AD is a chronic, progressive neurodegenerative condition marked by neuronal and synaptic degeneration in the cerebral cortex and subcortical regions. Early symptoms include memory decline, progressing to language impairment, emotional instability, among others. Pathologically, AD is marked by excessive amyloid β‐protein (Aβ) deposition and the formation of hyperphosphorylated tau protein [32]. Moreover, Aβ exhibits complex neurotoxicity, and studies using 3‐(4,5‐dimethylthiazol‐2‐yl)‐2,5‐diphenyltetrazolium bromide (MTT) assays have shown that oligomeric Aβ is more toxic than its monomeric form. In addition, primary hippocampal cell toxicity assays were used to investigate different forms of Aβ, including monomeric Aβ_1–40_, Aβ_1–42_, and oligomeric Aβ_1–40_, Aβ_1–42_. The results showed that oligomeric Aβ_1–40_ led to lower cell viability than monomeric Aβ_1–40_ but higher viability than oligomeric Aβ_1–42_, indicating that oligomeric Aβ_1–42_ is the most toxic. That being said, albiflorin reduced this toxicity and inhibited Aβ aggregation, providing neuroprotective effects [25]. Apart from Aβ, mitochondrial abnormalities are also closely related to AD. Mitochondrial dynamics disorders, including mitochondrial transport, autophagy, and the fission–fusion process, are associated with the development of NDDs [33]. Initially, mice were divided into four groups: wild‐type C57BL/6, APP/PS1 transgenic mice, and APP/PS1 mice treated with 20 or 40 mg/kg albiflorin. Transmission electron microscopy revealed that, compared to the wild‐type group, the APP/PS1 group exhibited an increased mitochondrial count and reduced mitochondrial length. However, albiflorin intervention with different dosages mitigated these changes by decreasing the mitochondrial count and increasing mitochondrial length [26]. Then, through a protein–protein interaction network, the molecular docking indicated a strong binding affinity between albiflorin and mitogen‐activated protein kinase‐1 (MAPK1) or extracellular signal‐regulated kinase (ERK), suggesting that these proteins may serve as potential targets for anti‐AD activity. In another in vitro experiment, an AD model was induced in PC12 cells using Aβ_25–35_. The model included a control group, a model group, and treatment groups with albiflorin at concentrations of 25, 50, 75, and 100 μM. MTT assay results showed reduced cell survival in the model group compared to the control, while all albiflorin‐treated groups showed significant improvement. Moreover, subsequent analyses indicated albiflorin could exert its effects by inhibiting PC12 cell apoptosis via the MAPK/ERK pathway, thereby offering potential therapeutic benefits for AD treatment [29].

Danggui‐Shaoyao‐San (DSS) was recorded as a classic prescription as early as the Han Dynasty in *Jin Gui Yao Lue* [34], with Shaoyao the Chinese name for *P. lactiflora* as the key component. In the 1980s, Nobuaki Mizushima, a Japanese scholar, proposed that DSS could be used to alleviate AD [35]. Subsequent studies further revealed that DSS could intervene in central nervous system diseases through its effects of anti‐inflammatory, antioxidant, neuroprotective properties, and by modulating neurotransmitters [36]. In essence, delving into the mechanisms through which the components of DSS contribute to the therapy of NDDs holds significant scientific and therapeutic value. Meanwhile, sAD has been found to be linked to oxidative stress and impaired calcium homeostasis. To investigate potential treatments, male C57BL/6 mice were divided into three groups: a sham group, a meningeal lymphatic vessel (MLV) ablation model group, and a treatment group receiving albiflorin at 28.8 mg/kg/day. Notably, immunofluorescence staining revealed a marked increase in IRF7‐positive cells in the hippocampus of the model group. However, this increase was significantly reduced in the intervention group. Furthermore, molecular docking analysis indicated that albiflorin might interact with IRF7, thereby enhancing brain lymphatic drainage and offering potential therapeutic benefits for sAD [28]. Additionally, JD‐30, an active fraction of the traditional Chinese formula DSS containing 26.69% albiflorin, was found to alleviate cognitive impairment in mice by reducing Aβ content and deposition. To further evaluate its effects, acute hippocampal slices were prepared from senescence‐accelerated mouse prone‐1 (SAMP1, control) and senescence‐accelerated mouse prone‐8 (SAMP8, model). The SAMP8 group received JD‐30 at 25, 50, or 100 mg/L, forming the SAMP1, SAMP8 + 25, SAMP8 + 50, and SAMP8 + 100 groups. Electrophysiological analysis demonstrated that JD‐30 reversed the reduction of long‐term potentiation in SAMP8 mice compared to SAMP1 controls, suggesting improved hippocampal neuronal function. Similarly, immunohistochemical staining showed elevated Aβ levels in SAMP8 mice relative to SAMP1, which were greatly reduced by JD‐30 treatment at 7, 14, and 28 mg/kg. These findings indicate that JD‐30 enhances neuronal activity and synaptic plasticity by decreasing Aβ levels and deposition [37]. Moreover, the neuroprotective effects of albiflorin, an active component in JD‐30, were extrapolated. In vitro experiments revealed that PC12 cell viability was improved with 50 μM albiflorin treatment compared to the Aβ_25–35_‐induced model group. In animal studies, Nissl staining showed that the P/A‐2 group, treated with a 1:8 (mg/kg) dose of albiflorin and paeoniflorin, exhibited significantly reduced hippocampal pyramidal neuron damage compared to the SAMP8 group. Additionally, the cellular layer structure remained mostly intact, and cell bodies were clearly visible [27].

### 2.2 Cerebral Ischemia

**FIGURE 1:**
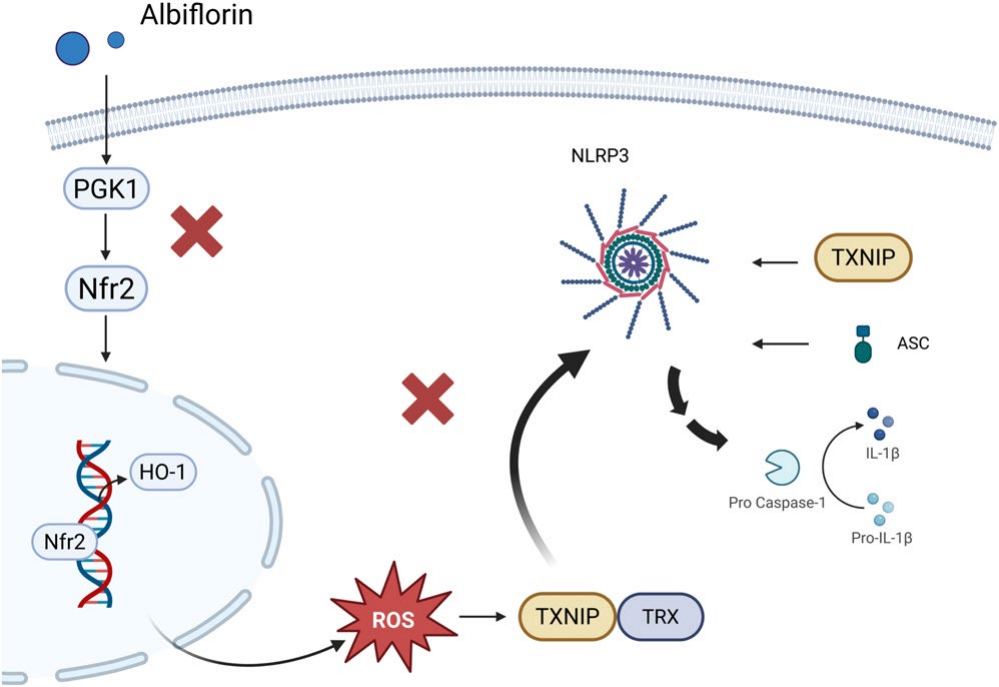
Graphic scheme of the potential role of albiflorin in alleviating neuroinflammation. Albiflorin inhibits PGK1 production and modulates the Nrf2/HO‐1 signaling pathway, reducing excessive ROS expression under pathological conditions. This ultimately suppresses the interaction of TXNIP with NLRP3 and ASC. Therefore, reducing pro‐caspase‐1 promotes the transformation of the inflammatory factor IL‐1β. ASC, apoptosis‐associated speck‐like protein containing a caspase recruitment domain; ROS, reactive oxygen species; TXNIP, thioredoxin interacting protein.

Cerebral ischemia (CI), which is caused by insufficient cerebral blood flow, leads to neuronal damage and cerebral infarction due to a lack of oxygen and essential nutrients. Among its types, ischemic stroke is the most common and significantly contributes to global mortality and disability, with neuroinflammation being key to its progression [38, 39]. In the context of traditional Chinese medicine, CI is often attributed to qi deficiency and blood stasis. Prolonged CI leads to irreversible necrosis of brain tissue, resulting in cerebral infarction. Moreover, this condition is often associated with a range of sophisticated physiological and pathological processes, such as inflammatory responses, blood–brain barrier (BBB) disruption, neuronal apoptosis, and oxidative stress [40, 41, 42]. Paeoniae Radix Rubra (Chi Shao) has long been used to clear heat, cool the blood, improve circulation, and relieve pain, often for ischemic cardiovascular and cerebrovascular diseases in China [43]. However, despite its known benefits, the intrinsic mechanism of action of albiflorin derived from Paeoniae Radix Rubra extract (PRRE) in the remedy of CI remains unclear. Histopathological analysis using hematoxylin–eosin (HE) staining demonstrated that PRRE protected against CI injury. Further serum analysis indicated that middle cerebral artery occlusion (MCAO) disrupted iron homeostasis in model rats, while PRRE treatment effectively reduced iron accumulation. Recent research highlighted the crucial role of glutathione peroxidase 4 (GPX4) in autophagy regulation and ferroptosis. In support of this, Western blotting (WB) revealed that PRRE treatment upregulated GPX4 and Beclin 1, a protein essential for autophagosome formation. To further explore its protective effects, hydrogen peroxide (H_2_O_2_) was applied to induce oxidative stress in HT22 cells, serving as the model group. Experimental groups included PRRE‐treated cells exposed to H_2_O_2_ and a combination group treated with PRRE and the phosphoinositide 3‐kinase/protein kinase B (PI3K/AKT) inhibitor LY249002 (PRRE + LY). Transmission electron microscopy analysis revealed that, compared to the model group with smaller and damaged mitochondrial structures, PRRE improved mitochondrial integrity and increased the accumulation of autophagosomes. However, these improvements were attenuated in the PRRE + LY group. Further molecular docking analysis suggested that albiflorin binds to Beclin 1 via hydrogen bonds, identifying it as a key component in PRRE to regulate ferroptosis and autophagy [15]. To assess the efficacy of albiflorin in cerebral infarction, rats were assigned to five groups: sham, MCAO, and treatment groups receiving low (5 mg/kg, L‐ALB), medium (10 mg/kg, M‐ALB), and high (20 mg/kg, H‐ALB) doses of albiflorin. After 7 days of treatment, WB analysis was performed on brain tissue samples to measure the expression of key proteins involved in the anti‐inflammatory process of albiflorin, including PGK1, thioredoxin‐interacting protein (TXNIP), and nucleotide‐binding oligomerization domain‐like receptor protein 3 (NLRP3). As expected, these protein levels were significantly higher in the MCAO group compared to the sham group, whereas albiflorin treatment dose‐dependently reduced these levels. Consistently, enzyme‐linked immunosorbent assay (ELISA) of prefrontal cortex and cerebrospinal fluid samples showed that albiflorin reduced inflammatory cytokines, including interleukin‐1β (IL‐1β), tumor necrosis factor‐α (TNF‐α), and interleukin‐6 (IL‐6), with the greatest reduction observed in the H‐ALB group. Furthermore, double immunofluorescence staining revealed colocalization of PGK1 with the microglial marker ionized calcium binding adaptor molecule 1 (Iba1) in the peri‐infarct region. Notably, MCAO increased PGK1 and Iba1 expression, both of which decreased with higher doses of albiflorin. These findings strongly suggest that albiflorin alleviates neuroinflammation and cellular damage after MCAO by reducing PGK1 levels, modulating inflammatory pathways, and potentially activating nuclear factor erythroid 2‐related factor 2 (Nrf2) nuclear translocation [14]. A graphical summary of these mechanisms is illustrated in Figure 1. Cerebral IR injury, which exacerbates brain damage during blood flow restoration post‐ischemia, poses a critical challenge in CI management. Nrf2, a key regulator of oxidative stress and inflammation, plays a protective role, while the antioxidant enzyme heme oxygenase 1 (HO‐1) maintains cellular homeostasis [44]. Recent studies proposed the Nrf2/HO‐1 signaling pathway as a promising therapeutic target for brain IR injury. Despite its potential, the precise mechanism by which albiflorin affects cerebral IR injury requires further investigation. Using quantitative real‐time PCR (qRT‐PCR), it was found that albiflorin at varying concentrations (0, 5, 10, and 20 μM) significantly reduced the mRNA expression levels of inflammatory cytokines, including *IL‐1β*, *IL‐6*, and *TNF‐α*, in oxygen–glucose deprivation/reoxygenation (OGD/R)‐induced PC12 cells. Additionally, WB analysis was conducted to assess the protein expression levels of Nrf2 and HO‐1 across different experimental groups, including the control group, OGD/R model group, and treatment groups administered albiflorin at concentrations of 0, 5, 10, and 20 μM [31]. In parallel, previous studies demonstrated that Chuanxiong‐Chishao elevated angiogenin‐1 (Ang‐1) levels in serum and increased the expression of hypoxia‐inducible factor‐1α (HIF‐1α) in the ischemic brain tissue of rats subjected to cerebral IR injury. These effects are likely associated with its ability to promote angiogenesis [45]. Building on this, computational network pharmacology and molecular docking techniques were employed to predict the therapeutic targets and signaling pathways of Chuanxiong‐Chishao in treating CI. The results revealed that albiflorin exhibited strong binding affinity with key targets, including MAPK1, steroid receptor coactivator, epidermal growth factor receptor, and caspase‐7. Binding affinity was assessed based on binding energy, with values below −5.0 kcal/mol indicating good binding, and below −7.0 kcal/mol indicating strong binding. Remarkably, the binding energies of albiflorin to the target proteins were −7.0, −7.8, −7.1, and −7.1 kcal/mol, respectively [30].

## 3 Neuropsychiatric Disorders (NPDs)

In addition to its therapeutic effects on the aforementioned NDDs, albiflorin, as a key active glycoside of *P. lactiflora* , also demonstrates significant efficacy in treating NPDs. Its mechanism of action is likely related to the regulation of the hypothalamic–pituitary–adrenal (HPA) axis and various central neurotransmitters, such as serotonin (5‐HT) and norepinephrine (NE) [46, 47]. Recent advances in understanding the gut microbiota also revealed multi‐pathway regulatory mechanisms of albiflorin in depression treatment. We presented the antidepressant effects of albiflorin and its underlying mechanisms in Table 2.

**TABLE 2:** The effect of albiflorin on NPDs and its possible mechanisms of action.

	Models	Mechanisms of action	References
Depression	Male ICR mice, male SD rats, male C57BL/6J mice and male Wistar rats	Albiflorin exerts its antidepressant effects by modulating hippocampal phospholipid and tryptophan metabolism, primarily by suppressing the overexpression of cytosolic phospholipase A2 (cPLA2)	[48]
Depression	SD rats	Albiflorin alleviates depression‐like behavior in model rats, possibly by regulating the neuroendocrine immune system and correcting disrupted metabolic pathways	[49]
Depression	Male SD rats	Albiflorin shows strong antidepressant effects by boosting hippocampal 5‐HT, NE, and brain‐derived neurotrophic factor (BDNF) levels	[50]
Depression	SD rats	Albiflorin exerts the effect of antidepressant to a chronic unpredictable mild stress (CUMS) rat model, involving the serotonergic and dopaminergic systems	[51]
Depression	Not known	Albiflorin may exert antidepressant‐like activity through targeting on mitochondrial metabolism	[52]
Depression	CUMS‐induced depression rats	Albiflorin mainly acts on synaptic function and neurotransmitter release in the hippocampus	[53]
Depression	SD rats	Albiflorin attenuates HPA axis hyperactivity, decreases serum nitric oxide (NO) and cyclic guanosine monophosphate (cGMP) levels, and inhibits the overexpression of serotonin receptor 2A (5‐HT2A) mRNA and protein	[54]
Depression	Male ICR mice and Wistar rats	Albiflorin exerts strong antidepressant‐like effects, primarily associated with elevated 5‐HT/NE levels and increased BDNF expression in the hippocampus	[55]
Depression	SD rats	Albiflorin's influence on the gut microbiota composition plays a positive role in alleviating depression	[21]
Post‐traumatic stress disorder	Male SD rats	Albiflorin decreases levels of allopregnanolone in prefrontal cortex, hippocampus, and reversed amygdala	[56]
Neuropathic pain	Male SD rats	Albiflorin may achieve alleviating effects by inhibiting the NLRP3 inflammasome and promoting the movement of Nrf2 into the nucleus, while also reducing nuclear factor‐kappa B (NF‐κB) activity in the hippocampus	[57]
Depression	PC 12 cells	Albiflorin exerts the effects on neuroinflammation by inhibiting the expression of NLRP3 protein and the activity of glutamine	[58]
Depression	ICR mice	The antidepressant effect of albiflorin is linked to the mitochondrial translocator protein 18 kDa (TSPO)	[59]
Depression	Male Kunming mice	The antidepressant mechanism of albiflorin may be related to its downregulation of the nitric oxide/cyclic guanosine monophosphate (NO/cGMP) pathway	[60]
Depression	Male SD rats	Albiflorin may exert its pharmacological effects by regulating monoamine neurotransmitters and cyclic nucleotides in the brain	[61]
Depression	Male Wistar rats	Albiflorin shows a clear antidepressant effect in olfactory bulbectomy (OBX) model rats, and its mechanism may be related to its ability to limit excessive activation of the HPA axis	[62]
Depression	Male SD rats	Albiflorin may exert its antidepressant effects by reducing HPA axis activation and modulating monoamine neurotransmitter levels in model rats	[63]
Depression	Male SD rats	Albiflorin may exert antidepressant effects by suppressing HPA axis activation and regulating abnormal levels of specific small molecule metabolites, such as phosphatidylcholine and carnitine derivatives, in model rats	[64]

**FIGURE 2:**
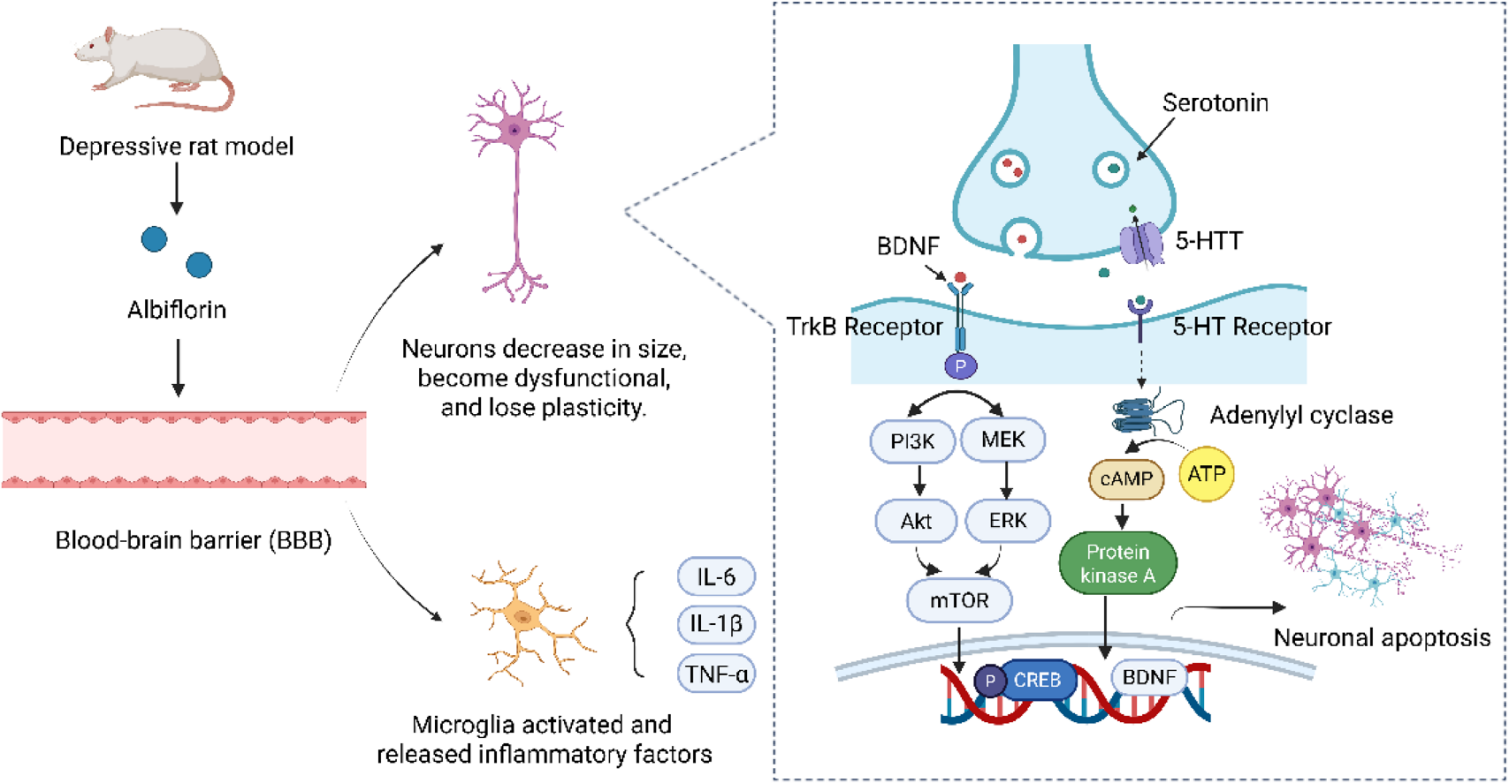
Graphic scheme of the role of albiflorin in alleviating depression. In a depressed rat model, albiflorin crosses the BBB, inhibits microglial activation, and reduces the expression of relevant inflammatory cytokines. Additionally, it promotes the release of BDNF through the PI3K/AKT, MEK/ERK, and cAMP/PKA signaling pathways, thereby aiding the recovery of damaged neurons. 5‐HTT, serotonin transporter; CREB, CAMP‐response element binding protein; MEK/ERK, mitogen‐activated extracellular signal‐regulated kinase/extracellular signal‐regulated kinase; TrkB, tropomyosin receptor kinase B.

Depression is a common mental disorder accompanied by feelings of sadness, impaired cognitive function, and notable changes in appetite or sleep patterns. Globally, approximately 300 million individuals are affected by varying degrees of depression, severely impacting daily life and making it one of the leading causes of disability worldwide. Although treatments for depression exist, more than 75% of individuals in low‐ and middle‐income countries do not receive adequate care due to factors such as high costs, limited therapeutic efficacy, and insufficient healthcare resources [65, 66]. Previous studies demonstrated that albiflorin could cross the BBB in vivo; however, its precise mechanism of action in curing depression is yet to be thoroughly investigated [67]. Exploring the mechanisms of albiflorin's antidepressant effects using in vivo and in vitro models is therefore of significance. As an example, different doses of albiflorin were administered to mice via gavage, followed by behavioral assessments. The results showed a significant reduction in immobility time in the forced swim test (FST) and tail suspension test (TST). Additionally, an open field test (OFT) was conducted in a rat chronic unpredictable stress (CUS) model to further validate these findings. WB analysis revealed that albiflorin treatment significantly increased BDNF expression in the hippocampus across all treated groups compared to the model group [55]. Further pharmacological experiments using CUMS, OBX, and LPS‐induced depression models were conducted to assess albiflorin's potential antidepressant effects via neuro‐metabolic pathways. Behavioral tests, such as TST, OFT, FST, and the sucrose preference test, were employed. Hippocampal tissues obtained from the CUMS depression models underwent HE and immunofluorescence staining to examine histopathological changes. The results indicated that albiflorin reduced pyramidal cell damage in the hippocampal CA3 region and counteract the decrease in proliferating cell nuclear antigen (PCNA)‐positive cells caused by CUMS. Additionally, WB analysis showed a notable increase in indoleamine 2,3‐dioxygenase 1 (IDO1), a crucial enzyme in tryptophan metabolism via the kynurenine pathway, in the hippocampus of CUMS‐induced depressive rats. Albiflorin treatment reduced IDO1 expression in a dose‐dependent manner [68]. Further in vitro tests demonstrated that albiflorin could inhibit cPLA2, a key enzyme in phospholipid metabolism, with an IC_50_ of 333.2 nM [48]. The antidepressant‐like effects of albiflorin were also examined in a chronic restraint stress‐induced rat model using behavioral tests such as the sucrose preference test. Compared with the model group, albiflorin increased the percentage of open arm entries (%OAE) to 36.21% and the percentage of time spent in the open arms (%TOA) to 15.12%. This result is comparable to fluoxetine, which achieved %OAE and %TOA values of 34.28% and 16.77%, respectively. Additionally, the levels of 5‐HT, 5‐hydroxyindoleacetic acid (5‐HIAA), NE, and dopamine (DA) in the hippocampus were measured. The results revealed that albiflorin significantly increased monoamine levels compared to the model group, including 5‐HT, 5‐HIAA, NE, and DA. Additionally, it upregulated neuronal nitric oxide synthase (*nNOS*) mRNA expression in the brain, though nNOS protein levels remained unchanged [54]. Mitochondrial dysfunction, alongside neurotransmitter imbalance, is increasingly acknowledged as a critical contributor in the development of depression. Mitochondrial translocator protein 18 kDa (TSPO), located in glial cells, facilitates cholesterol transport into mitochondria for neurosteroid synthesis. CUS was found to significantly reduce TSPO expression in the hippocampus of mice, while albiflorin treatment effectively restored its levels [59]. Albiflorin significantly reduced immobility time in the tail suspension test compared to the model group. Furthermore, different doses of albiflorin decreased NO and cGMP concentrations in the cerebral cortex. In the hippocampus, albiflorin treatment downregulated the mRNA expression of *nNOS*, soluble guanylate cyclase α2 subunit (*sGCα2*), and glutamate receptor 1 (*GluR1*) [60]. Albiflorin exerts antidepressant effects by modulating neurotransmitter and cGMP levels in rat model. Compared to the model group, albiflorin reduced cGMP levels and increased epinephrine, NE, DA, and 5‐HIAA. These findings further support albiflorin's potential in regulating neurotransmitter systems to alleviate depression [61]. To further investigate the effects of albiflorin on the HPA axis, an olfactory bulbectomy (OBX)‐induced depression rat model was used. Corticosterone (CORT) and adrenocorticotropic hormone (ACTH) levels in serum, as well as glucocorticoid receptor (GR) expression in the hippocampus, were measured in the OBX group and albiflorin‐treated groups. The results indicated that albiflorin reversed the OBX‐induced elevation of serum CORT and ACTH levels. Furthermore, GR expression in the hippocampus increased in the treatment groups compared to the model group, exhibiting a dose‐dependent trend [62]. Moreover, it was found that albiflorin's effects on the cyclic adenosine monophosphate/protein kinase A (cAMP/PKA) pathway in a rat model of blood deficiency and liver stagnation. WB analysis showed that compared to the model group, a 30 mg/kg dose of albiflorin significantly increased protein kinase A (PKA) levels in the cortex and hippocampus [63]. In summary, albiflorin exerts its antidepressant effects through multi‐target mechanisms, including the regulation of monoamine neurotransmitter levels, promotion of BDNF expression, inhibition of neuroinflammation, and modulation of HPA axis function. By improving the pathophysiological state of depression through these mechanisms, albiflorin demonstrates promising potential for clinical application. The pharmacological mechanisms by which albiflorin exerts its effects in the brains of depressed rat models after intragastric administration is illustrated in Figure 2.

## 4 Other Nervous System Diseases

In addition to its effects on mental disorders like AD and depression, albiflorin also demonstrated therapeutic potential in other neurological conditions, including stroke, convulsions, and hypoxic–ischemic brain injury [69, 70, 71, 72]. However, pharmacological studies on albiflorin's effects in these diseases remain limited. We summarize available literature in Table 3.

**TABLE 3:** The effect of albiflorin on other nervous system diseases and its possible mechanisms of action.

	Models	Mechanisms of action	References
Brain stroke	Pharmacophore models	Albiflorin's effectiveness may be linked to the platelet activating factor receptor, angiotensin‐converting enzyme (ACE), and 5‐HT2A	[70]
Convulsions	Male Wistar rats	Albiflorin could exert its anticonvulsant effect by inhibiting the seizure‐induced reduction of extracellular calcium, thereby preventing the subsequent increase in intracellular calcium levels	[71]
Hypoxic ischemic brain injury (HIE)	The HIE model of neonatal mice	Albiflorin reduces cerebral infarction, inhibits brain cell apoptosis, oxidative stress, and inflammation, and protects against nerve injury, possibly through activating the phosphatidylinositol 3‐kinase/protein kinase B/mammalian target of the rapamycin (PI3K/Akt/mTOR) signaling pathway	[72]

Brain stroke occurs when the blood supply to the brain is interrupted or reduced, leading to oxygen and nutrient deprivation and causing damage or death of brain cells. It is primarily classified into ischemic stroke and hemorrhagic stroke [73]. Among traditional treatments, Huatuo Reconstruction Pill (HTRP) is a classical and historic Chinese herbal formula commonly used in clinical practice to alleviate stroke symptoms. Modern pharmacological studies have not only demonstrated its antithrombotic effects but also highlighted its protective role in stroke. By screening the active components of HTRP to explore its therapeutic targets, it was found that albiflorin interacts with the pharmacophore model of ACE. These finding suggest that albiflorin might be a key therapeutic agent within HTRP, contributing to its efficacy in disease treatment. However, this finding has not yet been experimentally validated using pharmacological methods [70]. Furthermore, the anticonvulsant effects of peony root appear to be closely related to albiflorin, as evidenced by its impact on electroencephalography power spectrum changes and extracellular calcium and potassium ion concentration variations in pentylenetetrazol‐induced rats. Compared to the model group (S/F value: 1.85 ± 0.18), albiflorin (25 mg/kg) notably reduced electroencephalography power spectrum alterations, with an S/F value of 1.11, and fully prevented changes in extracellular calcium and potassium ion concentrations [71]. HIE in the perinatal period is a common form of brain injury caused by factors such as neonatal asphyxia, leading to cerebral hypoxia or ischemia. Clinically, it manifests as altered consciousness and muscle tone abnormalities, with underlying pathological processes involving neuronal damage and inflammation. Recent pharmacodynamic studies highlighted the therapeutic potential of albiflorin in neurological injury treatment. In a neonatal mouse HIE model, 2,3,5‐triphenyltetrazolium chloride (TTC) staining revealed that cerebral infarction rates were significantly lower in the albiflorin‐treated group compared to the model group. WB analysis confirmed the presence of cleaved caspase‐3 and caspase‐9 in brain cells, while ELISA assays indicated significantly lowered levels of inflammatory cytokines including interleukin (IL)‐6, IL‐9, and IL‐1β. Additionally, analysis of the PI3K/AKT/mTOR signaling pathway showed a decrease in phosphorylated PI3K, AKT, and mTOR by 40%, 55.56%, and 49.31%, respectively, in HIE mice. However, albiflorin treatment partially restored their phosphorylation levels [72]. In addition to these findings, albiflorin was identified as an active component in certain traditional Chinese medicine formulas [74, 75, 76]. Despite this growing body of evidence, the precise mechanisms underlying albiflorin's therapeutic effects remain poorly understood. Therefore, further investigations are warranted to elucidate its actions and establish a robust foundation for possible clinical applications.

## 5 Metabolism and Distribution in Brain

Although extensive research was conducted for a better understanding of the therapeutic effects and underlying mechanisms of albiflorin in mental disorders, studies integrating its metabolic profile and tissue distribution remain limited. By analyzing hippocampal metabolic changes in three depression models (CUMS, OBX, LPS‐induced rat model) from both lipidomic and metabolomic perspectives, researchers identified four key metabolic pathways: phospholipid metabolism, arachidonic acid metabolism, tryptophan metabolism, and the tricarboxylic acid (TCA) cycle. Furthermore, a metabolomics analysis highlighted the top 20 hippocampal metabolites linked to increased locomotor activity after albiflorin treatment, showing its antidepressant effects by restoring phospholipid and tryptophan metabolism. Additionally, by inhibiting cPLA2, albiflorin corrects metabolic dysregulation in depression. Pharmacokinetic analysis revealed that albiflorin was detectable in the plasma and hippocampus within 5 min, with peak levels reached at 30 min in plasma and 45 min in the hippocampus [48]. Moreover, when administered separately, albiflorin and saikosaponin A improved mitochondrial function by regulating the TCA cycle (reducing the levels of succinate and l‐malic acid) and inhibiting NLRP3 protein expression to alleviate the inflammatory response in PC12 cells under depressive pathological conditions, exerting a synergistic neuroprotective effect. Notably, their combined use demonstrated superior efficacy compared to individual administration [58]. Similarly, another study demonstrated significant regulatory effects of albiflorin in a depression model induced by radiation combined with chronic restraint stress and isolation, based on behavioral characteristics, peripheral blood parameters, organ indices, and HPA axis‐related pharmacodynamic indicators. Peripheral blood analysis revealed an upward trend in white blood cell, red blood cell, and hemoglobin levels in the albiflorin‐treated group compared to the model group. Furthermore, metabolomic analysis of serum and cortical samples showed that albiflorin promoted the recovery of abnormal biomarkers, such as phosphatidylcholine and lysophosphatidylcholine, while modulating multiple metabolic pathways, including ether lipids, glutamate, and arachidonic acid [64]. Additionally, a competitive binding assay was performed in rat brain tissue to evaluate the interaction between albiflorin and monoamine transporters, comparing its effects with those of fluoxetine (a selective serotonin reuptake inhibitor) and desipramine (a norepinephrine reuptake inhibitor). The results showed that the inhibition constant (*K*_i_) values for albiflorin were 5.25 ± 0.17, compared to 16.32 ± 1.02 for fluoxetine, and 1.25 ± 0.77, compared to 7.38 ± 0.67 for desipramine. Furthermore, microdialysis was used to measure extracellular 5‐HT and NE levels in the hypothalamus of freely moving rats. Following albiflorin administration, both 5‐HT and NE levels showed an increasing trend, peaking at 120 min. In addition to investigating albiflorin as a standalone compound, its antidepressant effects have also been explored within traditional Chinese medicine formulations [50]. For example, serum pharmacochemistry and network pharmacology approaches were used to investigate the antidepressant effects of Chaihu Shugan San (CHSGS). In this study, rats were administered CHSGS at a dose of 30 g/kg via gavage, and the active component albiflorin was analyzed in plasma. The pharmacokinetic parameters of albiflorin were determined as follows: biological half‐life (*T*_1/2_) of 12.10 ± 1.24 h, time to maximum concentration (*T*_max_) of 0.46 ± 0.10 h, maximum concentration (*C*_max_) of 575.95 ± 292.48 ng/mL, area under the plasma concentration–time curve from zero to the last measurable concentration (AUC_last_) of 1477.44 ± 210.69 ng·h/mL, and total area under the curve (AUC_0–∞_) of 2877.09 ± 392.95 ng·h/mL [53].

**FIGURE 3:**
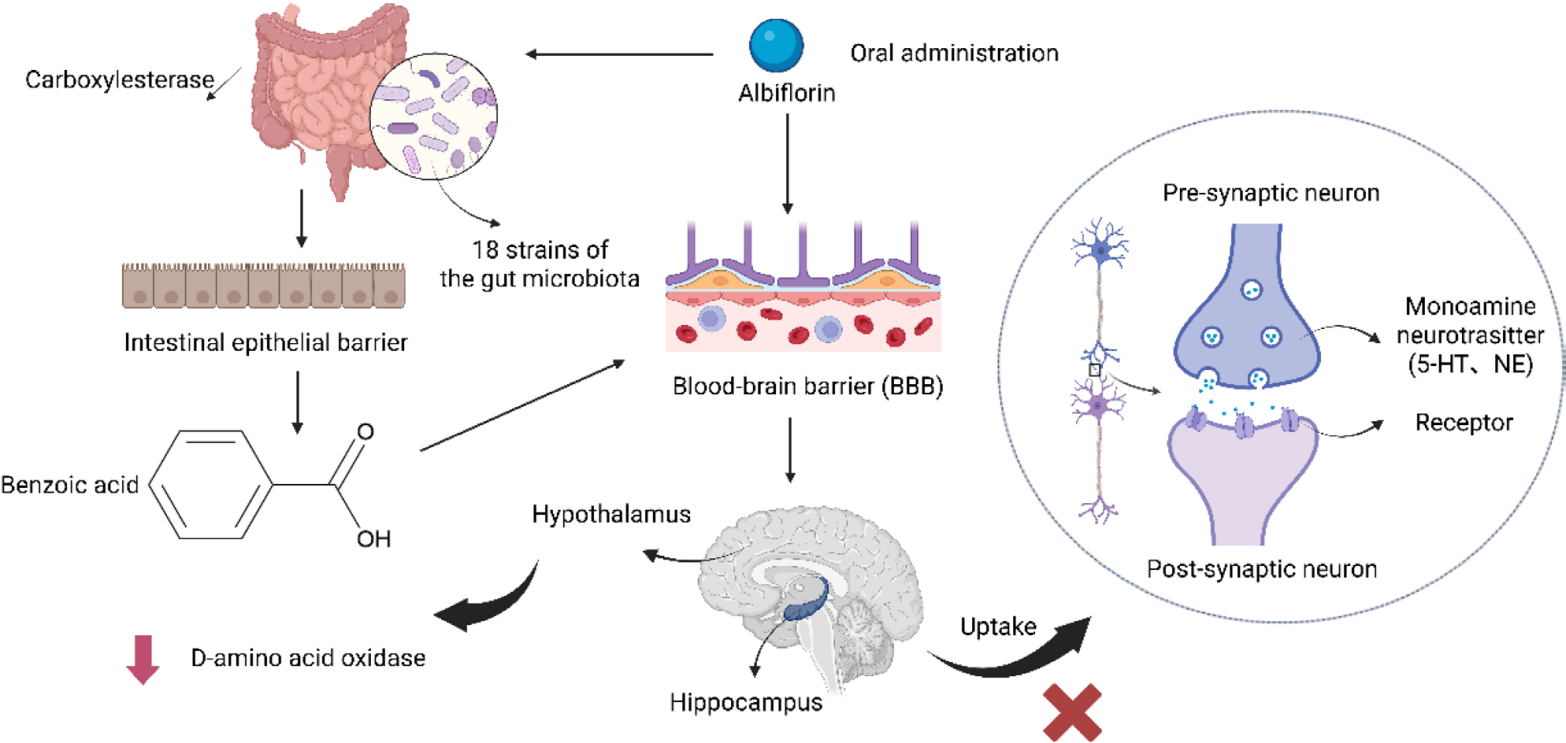
Graphic scheme of distribution of albiflorin. Albiflorin is administered orally and enters the body, with one portion directly accessing the brain via the BBB. Another portion is metabolized in the gut by 18 types of gut microbes and carboxylesterases, converting into benzoic acid, which then enters the brain. Ultimately, this process influences the formation of d‐amino acid oxidase in the hypothalamus and the uptake of neurotransmitters (5‐HT, NE) in the hippocampus.

Despite these insights, pharmacokinetic studies on albiflorin have been limited, possibly due to the difficulty in detecting its extremely low levels in the brain as the prototype compound. Nevertheless, it was confirmed that albiflorin is capable of crossing the BBB [67, 77]. Although albiflorin is a natural antidepressant with a favorable safety profile, its brain concentration remains low and challenging to detect after oral administration. Given the increasing focus on the gut microbiota's role in brain function, it is worthwhile to test whether albiflorin's antidepressant effects in vivo are mediated by the gut microbiota. Researchers first cultured rat gut microbiota in vitro and identified benzoic acid (BA) as the primary metabolite of albiflorin. Notably, BA was found to cross the blood–brain barrier (BBB) and reach the central nervous system to possibly elevate d‐serine levels. Furthermore, BA concentrations in plasma and brain tissue were measured in normal and pseudo‐germ‐free (PGF) mice following oral administration of albiflorin. In normal mice, the brain *C*_max_ and AUC_0–*t*_ of BA were 2.1‐ and 2.2‐fold higher, respectively, than in PGF mice, while in plasma, *C*_max_ and AUC_0–*t*_ were 1.73‐ and 1.74‐fold higher. However, after intravenous administration of 1.75 mg/kg albiflorin, plasma concentrations were nearly undetectable within 2 h [21]. In summary, as a natural compound, the pharmacokinetics of albiflorin remain underexplored, particularly regarding its brain distribution and ability to effectively cross the BBB, which warrants further investigation. Based on current literature, we propose the distribution of albiflorin in vivo in Figure 3.

## 6 Toxicity

Despite toxicological reports on albiflorin remaining limited, a handful of in vivo and in vitro studies are available for reference [78]. In animal models, albiflorin is typically administered orally or intraperitoneally at doses ranging from 5 to 200 mg/kg [14, 19, 26]. In vitro studies have shown that albiflorin exhibits no significant cytotoxicity in various cell lines (like PC 12 cells) at concentrations of 1–100 μM, with cell viability remaining above 90% [58, 79]. Han et al. conducted a series of conventional toxicity assessments, including acute oral toxicity tests in rats and dogs, in vitro mammalian chromosomal aberration assays, and bone marrow micronucleus tests. The results indicated that even high doses of albiflorin (up to 5000 mg/kg) did not produce observable toxic effects. To further investigate the possibility of subtle or delayed toxic effects, next‐generation metabolomics was employed in a rat model treated with 7 mg/kg albiflorin. Analysis of urinary metabolites revealed no significant alterations among the top 30 differential metabolites, as confirmed by MetaboAnalyst 3.0 and disease‐related metabolite databases, suggesting no detectable metabolic toxicity [52]. However, according to current literature, data on albiflorin from non‐human primate models and long‐term toxicological studies are still lacking. Therefore, further research in these areas is needed to comprehensively evaluate and confirm the safety profile of albiflorin.

## 7 Conclusion

In summary, albiflorin is a key bioactive component in *P. lactiflora* , which demonstrates therapeutic potential through its multitarget regulatory mechanisms, particularly in the intervention of mental disorders including AD and depression. Albiflorin's effects on mental disorders emphasize its regulatory capabilities in multiple aspects, including its pharmacokinetics, mechanisms of action, and tissue distribution, as revealed by in vivo and in vitro studies. Current pharmacological research suggests that albiflorin's primary mechanisms in treating mental disorders involve modulating monoamine neurotransmitter levels and inhibiting neuroinflammation. However, certain aspects require further investigation, including its specific regulatory pathways through the gut–brain axis, the optimization of effective brain concentrations in animal models, and its synergistic mechanisms within traditional Chinese medicine formulations. Although albiflorin shows great therapeutic potential, transforming it from a natural compound into a standardized modern drug still faces several critical challenges. Future research should focus on elucidating its mechanisms of action using complex disease models, identifying active metabolites and specific molecular targets. Given the limited brain distribution of albiflorin, formulation strategies such as nanotechnology may help improve its bioavailability, enhance solubility, and maintain effective plasma concentrations. High‐quality clinical trials of albiflorin as a single compound are also essential. These should not only generate robust human data but also rigorously assess potential pharmacokinetic interactions with commonly used medications to ensure safety. It is precisely the resolution of these issues that is essential for the clinical advancement of albiflorin. With future advancements integrating cutting‐edge pharmacological approaches, metabolomics, and clinical studies, albiflorin holds promise as an effective strategy for the treatment of mental disorders.

## Author Contributions

**Shasha Sun:** formal analysis, methodology, writing – original draft. **Hamizah Shahirah Hamezah:** investigation. **Chuanshan Jin:** funding acquisition, investigation. **Rongchun Han:** formal analysis, writing – original draft. **Xiaohui Tong:** conceptualization, project administration, writing – review and editing.

## Conflicts of Interest

The authors declare no conflicts of interest.

## Data Availability

The data that support the findings of this study are available from the corresponding author upon reasonable request.
